# Spatial Patterns and Temperature Predictions of Tuna Fatty Acids: Tracing Essential Nutrients and Changes in Primary Producers

**DOI:** 10.1371/journal.pone.0131598

**Published:** 2015-07-02

**Authors:** Heidi R. Pethybridge, Christopher C. Parrish, John Morrongiello, Jock W. Young, Jessica H. Farley, Rasanthi M. Gunasekera, Peter D. Nichols

**Affiliations:** 1 CSIRO Oceans and Atmosphere Flagship, Hobart, Tasmania, Australia; 2 Department of Ocean Science, Memorial University of Newfoundland, St. John's, Newfoundland, Canada; 3 School of BioSciences, University of Melbourne, Parkville, Victoria, Australia; 4 CSIRO Food, Nutrition and Bioproducts Flagship, Hobart, Tasmania, Australia; Institute of Marine Research, NORWAY

## Abstract

Fatty acids are among the least understood nutrients in marine environments, despite their profile as key energy components of food webs and that they are essential to all life forms. Presented here is a novel approach to predict the spatial-temporal distributions of fatty acids in marine resources using generalized additive mixed models. Fatty acid tracers (FAT) of key primary producers, nutritional condition indices and concentrations of two essential long-chain (≥C_20_) omega-3 fatty acids (EFA) measured in muscle of albacore tuna, *Thunnus alalunga*, sampled in the south-west Pacific Ocean were response variables. Predictive variables were: location, time, sea surface temperature (SST) and chlorophyll-a (Chla), and phytoplankton biomass at time of catch and curved fork length. The best model fit for all fatty acid parameters included fish length and SST. The first oceanographic contour maps of EFA and FAT (FATscapes) were produced and demonstrated clear geographical gradients in the study region. Predicted changes in all fatty acid parameters reflected shifts in the size-structure of dominant primary producers. Model projections show that the supply and availability of EFA are likely to be negatively affected by increases in SST especially in temperate waters where a 12% reduction in both total fatty acid content and EFA proportions are predicted. Such changes will have large implications for the availability of energy and associated health benefits to high-order consumers. Results convey new concerns on impacts of projected climate change on fish-derived EFA in marine systems.

## Introduction

Assessing the spatial and temporal distribution and availability of essential nutrients and energy is vital to understanding what drives or constrains ecosystem functions and services. It is also fundamental to human nutrition and food security. Fatty acids are constituents of lipids and are essential for adequate nutrition, health, development and growth of all organisms [1, 2], including humans where the marine-derived omega-3 long-chain (≥C_20_) polyunsaturated fatty acids (omega-3 or ɷ3 PUFA) are a major contributor to brain development [3]. Among other crucial functions, fatty acids maintain the structural integrity, viability and function of cells, and regulate gene expression, ion balance and hormones [4, 5].

In marine organisms, about twenty different fatty acids are present at relative amounts of greater than one percent. The relative composition of different fatty acids can vary considerably within and between individuals, species and populations. This can be due to influences of biological (tissue type, length, age, maturity) and environmental (e.g. diet, water temperature, productivity) features, in addition to genetics [6, 7]. In marine environments, certain fatty acids, particularly LC-PUFA, can only be incorporated in the food web after they have been synthesized by primary producers such as phytoplankton or macroalgae [8] or certain bacteria [9]. Different taxonomic and functional groups (e.g. diatoms, dinoflagellates, macroalgae) have unique fatty acid signatures [10] that can be used as fatty acid trophic markers or tracers (FAT) to provide novel insight into energy and nutrient transfer from lower producers to higher order consumers [11].

Despite mounting evidence of climate-induced changes in phytoplankton phenology, distribution, species composition and abundance [12–14], the potential consequences of these ecosystem services and food security are relatively unknown. It is essential to develop methods that can detect temporal and spatial shifts between key functional groups of primary producers. This is because such shifts directly influence the structure and function of ecosystems by altering the form and availability of essential fatty acids, and other key nutrients. Indeed, shifts in primary producers will greatly affect energy transfer efficiencies [15] which is likely to alter the supply of essential fatty acids to higher order consumers [16, 17]. While several studies have described temporal changes in phytoplankton-derived essential fatty acids [18–20], few have attempted to understand the major intrinsic and external drivers so that future predictions can be made.

This study creates a novel framework to quantify spatial and temporal changes in essential and biomarker fatty acids measured in the muscle of albacore tuna, *Thunnus alalunga*, sampled across the south-west Pacific Ocean. Albacore was an ideal study species as it is a top-predator that provides significant economic and nutritional benefits to humans and has a broad worldwide distribution from tropical and temperate waters. Furthermore, this study follows on from previous fatty acid and stable isotope work on albacore tuna in the region that showed clear geographic differences that are likely related to known environmental features in the region [21, 22]. The study area has undergone considerable recent environmental change, with the major oceanic feature, the East Australian Current, extending further south [23, 24] which in turn has resulted in increases in temperature, salinity and nitrate and a decline in silicate [25]. Such changes are expected to induce long-term latitudinal shifts in phytoplankton species and size composition, as has been shown in other climate ‘hotpot’ areas of the world [26]. Through detailing the distribution of fatty acids assimilated in tuna muscle, our study provides spatially and temporally resolved trophic structures and availability of essential fatty acids to humans through seafood consumption across the south-west Pacific. We also consider the role of essential fatty acids in ecosystem stability and energy pathways.

## Methods

### Ethics statement

No field permits or ethical approvals were required for this study, as all fish originated from commercial or recreational fisheries and were already dead when provided to the sampler. No samples were collected by the authors. Fish were sacrificed by the commercial or recreational fishers at sea using standard fisheries practices (most fish were dead when landed). Permission was granted to use samples from all fish. All samples were donated. Albacore tuna are not a protected species in any ocean.

### Study species and sampling

We sampled muscle tissue of 154 albacore caught at 29 distinct sampling locations (with coordinates rounded to the third decimal degree) along the eastern coast of Australia and off the south-west coast of New Zealand, between January and December in 2009 and 2010 (data in S1 and S2 Tables). In Australia, samples were obtained from long-line catches in Queensland and New South Wales; from recreational catches in the west Tasman Sea off Tasmania; and from the domestic troll fishery in New Zealand. Coordinates were taken from fishing vessels within two hours of the time of sampling. Samples collected from all regions were collected either by observers on long-line fishing vessels, from scientist at sea during tagging operations, or directly by the fishing crew. Fork length (FL) of each fish was measured to the nearest cm and a sample of flesh was taken from the back of the head and frozen at -20°C until lipid analysis.

Satellite-derived sea surface temperature (SST,°C) and sea surface chlorophyll-*a* concentration (Chl*a*, mg m^-3^) data were obtained using the spatial dynamics ocean data explorer (SDODE) interface [27] for each sampling time (day in year) and location (latitude and longitude) at a resolution of 0.036 x 0.042° lat. x long (~ pixel size of 4 x 4 km). SST data were composed using moving average intervals of a 3 and 15 day composite period based on pathfinder (SST_3_ or SST_15_). Chl*a* data were derived from MODIS over an 8 and 30 day composite period (Chl*a*_8_ or Chl*a*_30_ *)*. Median phytoplankton cell mass (M_B50_) was estimated using SST_3_ and Chl*a*_8_ data and equations based on Barnes et al. [28]: M_B50_(log_10_ pg C) = 1.340–0.043(SST) + 0.929(Log_10_(Chl*a*)).

### Fatty acid analysis

Procedures used for direct transmethylation of wet tissue were based on those described by Parrish et al. [29]. Wet fish muscle tissue (0.04–0.05 g) was ground, weighed and directly transmethylated in MeOH:CHCl_3_:HCl (10:1:1 v/v) for 2 hours at 80°C. After cooling, 1.5 ml of Milli-Q water and a known concentration of internal injection standard (23:0 FAME) were added in 1.5 ml of hexane, followed by 0.3 ml of dichloromethane. Samples were centrifuged and the upper, organic layer was removed under a nitrogen stream. After the addition of chloroform, samples were injected into the gas chromatograph (GC) equipped with a non-polar Equity-1 fused silica capillary column, a flame ionization detector, a split/splitless injector and an Agilent Technologies 7683B Series autosampler. Peaks were verified using a Finnigan Thermoquest GCQ GC/MS and were quantified using Agilent Technologies ChemStation software (Palo Alto, California USA).

We used known fatty acid tracers (FAT) based on relative proportions of individual or summed fatty acids that are characteristic of marine primary producers [11, 30]: diatoms (EPA + 14:0); dinoflagellates (DHA); green algae, cryptophytes and macroalgae (C_18_PUFA algae = 18:2ɷ6 + 18:4ɷ3 + 18:3ɷ3), and grazing and detritivorous primary consumers (ɷ6 LC-PUFA protists = 20:4ɷ6 + 22:5ɷ6 + 22:4ɷ6). Potential primary producers of C_18_ PUFA include cryptophytes which are characteristically high in 18:2ɷ6 [31] and brown algae (phaeophyta) high in 18:4ɷ3 [11]. The ɷ6 LC-PUFA are connected through the fatty acid synthase or the polyketide synthase pathways [32]. The 20:4ɷ6 has also been associated with benthic protists [33] and rhodophytes (red algae: [11]). The detritivores thraustochytrids and labyrinthulids have been associated with 22:5ɷ6 and 22:4ɷ6 [34]. For all fatty acids that were summed, significant Spearman correlations (R^2^> 0.90, p<0.001) between individual fatty acids were observed, and individually they had all previously been associated with the spatial distribution of albacore tuna fatty acids [21]. For example, the vectors for 20:4ɷ6, DHA, and 14:0 had the strongest Pearson correlations in a principal component analysis (PCA) of albacore tuna fatty acids.

Total fatty acid content (TFA, % of total mass), which has a positive linear correlation with total lipid content in albacore muscle (R^2^ = 0.97, *p*<0.001, [30]) was used as a nutritional condition index (NCI) with higher values associated with larger energy reserves and better health [8]. Calculated ratios of omega-3 versus omega-6 polyunsaturated fatty acids (ɷ3/ɷ6) were also used as an index of fish nutritional condition with higher values indicative of a fish in better nutritional condition with respect to human consumption [35]. TFA and concentrations of essential fatty acids (EFA) EPA and DHA were calculated using known concentrations of internal standard solution.

### The models

Generalized additive mixed models (GAMMs; [36]) were generated to describe and predict spatiotemporal patterns of essential and biomarker fatty acids. Different GAMMs were run to test the effect of several non-linear covariates: fork length, spatial (latitude and longitude), environments (SST, Chl*a*, or M_B50_) and time (day of year). Multiple fish were collected from each localized sampling location; these were aggregated into one of 10 regions based on proximity of sampling location (latitude and longitude) (data in S2 Table). Region was included as a random intercept in models to reflect the data’s underlying hierarchical nature and account for geographic variation not explained by the main effects. Fork length was included in all models to account for intrinsic physiological processes and a potentially confounding spatial distribution pattern; large individuals are caught more often in the north and smaller individuals in the south of the study area. With the exception of model 12, environmental variables, SST and Chl*a*, were separated due to colinearity (R^2^ = 0.46, *p* = 0.007). Data for FAT, EFA and TFA content were log_10_ transformed to meet model assumptions of homogeneity of variance. Ratios of ɷ3/ɷ6 did not require transformation prior to analysis.

Model performance was evaluated using standard diagnostic checks. Model selection (data in S3 Table) was based on minimization of the corrected (for small sample size) Akaike information criteria (AICc, [37]). Model performance was evaluated by cross-validation where the full model was plotted against model predictions for an independent dataset [21]. Predictions based on the GAMMs were made using least square regression analysis of particular sections of the smoothing spline (e.g. <18°C or M_B50_ (log_10_) < or > 0). Analyses were performed using the gamm4 package [38] in R [39].

### FATscapes

Based on a GAMM including sampling location (latitude, longitude) as the only predicative variable (model 4), oceanographic spatial contour maps (here named FATscapes as an extension to the well known isoscapes: [40]) were produced to graphically illustrate the predicted and interpolated distribution of fatty acid parameters measured in albacore tuna muscle tissue. FATscapes were used to delineate and define geographically distinct fatty acid bioregions.

## Results

Sixty-six fatty acids were identified of which relative proportions of the top 11 are presented in S1 Table. Spearman’s ranked correlation showed highly significant correlations between FAT of diatoms and C_18_PUFA synthesizing algae (C_18_ algae: R^2^ = 0.91, p<0.001) and between FAT of dinoflagellates and ɷ6 LC-PUFA synthesizing protists (ɷ6 protists: R^2^ = 0.78, p = 0.008). Model formulations for each FAT, EFA (mg/100 g), and NCI’S, including TFA (as % of wet tissue) and ratios of ɷ3/ɷ6 PUFA are given in S3 Table, with the best model for the eight response variables presented in Table 1. Fork length and SST_3_ were included in five of the eight best models. The three exceptions were for FAT of ɷ6 protists which included fork length and median phytoplankton cell mass, and for TFA and DHA which included fork length only (Table 1). Geographical location (Latitude, Longitude), day of year (DOY) or satellite-derived observations of Chl*a* proved to be less efficient predictors for FAT and EFA.

**Table 1:** Parametric coefficients for the best model fits for environmental and geographical models predicting fatty acids tracers (FAT), nutritional condition indices (NCI), and essential fatty acid concentrations (EFA) as determined by AICc (S3 Table). FAT are divided into 3 groups of primary producers (diatoms, dinoflagellates, C_18_ PUFA producing algae) and a group of grazing and detritivorous primary consumers (LC-ω6 PUFA producing protists). FL—fork length; SST_3_ and SST_15_ –sea surface temperature of 3 and 15 day composites, respectively; M_B50_ –median phytoplankton cell mass. Edf—equivalent degrees of freedom; ɷ –omega; PUFA—polyunsaturated fatty acids; LC—long-chain (≥C_20_), TFA—total fatty acid (mass %).

		Best model	Intercept	Edf	F-value	p-value	DE%
**FAT**	Diatoms: log(EPA%+14:0%)	~s(FL)	1.79±0.04	2.75	2.75	0.01*	67
		+s(SST_3_)		3.58	3.58	3.07e^-07^ ***	
	Dinoflagellates: log(DHA%)	~s(FL)	3.27±0.04	2.92	8.79	2.81e^-05^ ***	42
		+s(SST_3_)		3.72	11.03	2.05e^-07^ ***	
	C_18_ algae: log(ΣC_18_ PUFA%)	~s(FL)	0.37±0.05	3.14	5.85	7.25 e^-04^ ***	74
		+s(SST_3_)		3.39	7.66	4.71e^-05^ ***	
	ɷ6 protists: log(ΣLC-ω6 PUFA%)	~s(FL)	1.26±0.05	3.54	12.21	7.77e^-08^ ***	72
		+s(M_B50_)		3.46	5.66	6.57 e^-04^ ***	
**NCI**	Σɷ3/Σɷ6 PUFA	~S(FL)	7.14±0.10	3.42	40.25	<2.00e^-16^ ***	71
		+s(SST_3_)		3.2	6.81	1.03e^-04^ ***	
	log(TFA%)	~S(FL)	0.45±0.08	2.68	1.73	0.02*	60
**EFA**	log(EPA mg/100 g)	~S(FL)	3.04±0.09	2.90	3.07	0.03*	66
		+s(SST_15_)		1.00	7.59	6.57e^-03^ **	
	log(DHA mg/100 g)	~S(FL)	4.99±0.08	1.32	3.96	0.04*	50

* Significant,

** Moderately significant,

*** Highly significant.

**Fig 1:**
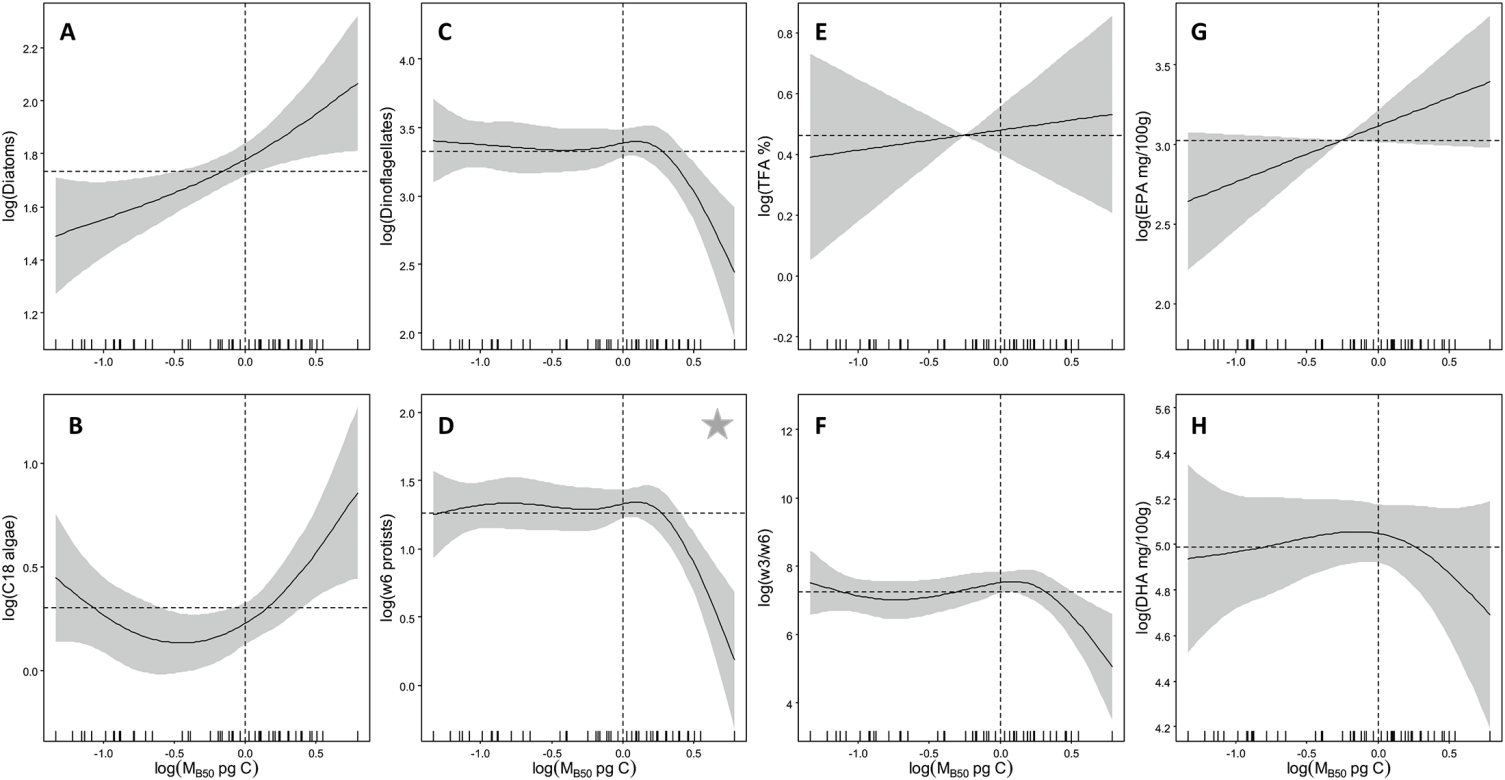
Relationship between observed median phytoplankton cell mass and GAMM predicted fatty acid parameters in albacore. Fatty acid parameters include those representative of: (A) diatoms, (B) C_18_ algae, (C) dinoflagellates, (D) ɷ6 protists, (E) total fatty acid content (% of tissue), (F) ratios of ɷ3/ɷ6, (G) EPA, and (H) DHA. The dashed horizontal black lines represent the intercept (or zero line) in each plot. The dashed vertical lines approximately delineate the range of the explanatory variable above the zero line used as thresholds. The solid grey area bracketing the response curves show the confidence limits of the model and are twice the standard error. A star represents the model fit for that particular response variable with the highest % deviance explained (S3 Table).

Changes in median phytoplankton cell mass [M_B50_(log_10_ pg C)] were reflected in FAT of all primary producers, EFA concentrations, and to a lesser extent in NCI (Fig 1). Model plots of the FAT- M_B50_ relationship show clear transitions at M_B50_ of 0 (or 1.0 pg C as an inverse logarithm) with diatoms increasing almost linearly, while C_18_ algae showed a compound curve with highest proportions found in largest values of M_B50_. FAT of dinoflagellates and ɷ6 protists showed limited variation < 1.0 M_B50_ pg C, but sharply declined thereafter. Relationships between EFA concentrations and M_B50_ were similar to those observed for diatoms (EPA) and dinoflagellates (DHA). Opposite trends between the two NCI and M_B50_ were observed with proportions of TFA increasing, and ratios of ɷ3/ɷ6 PUFA decreasing with increasing M_B50_.

**Fig 2:**
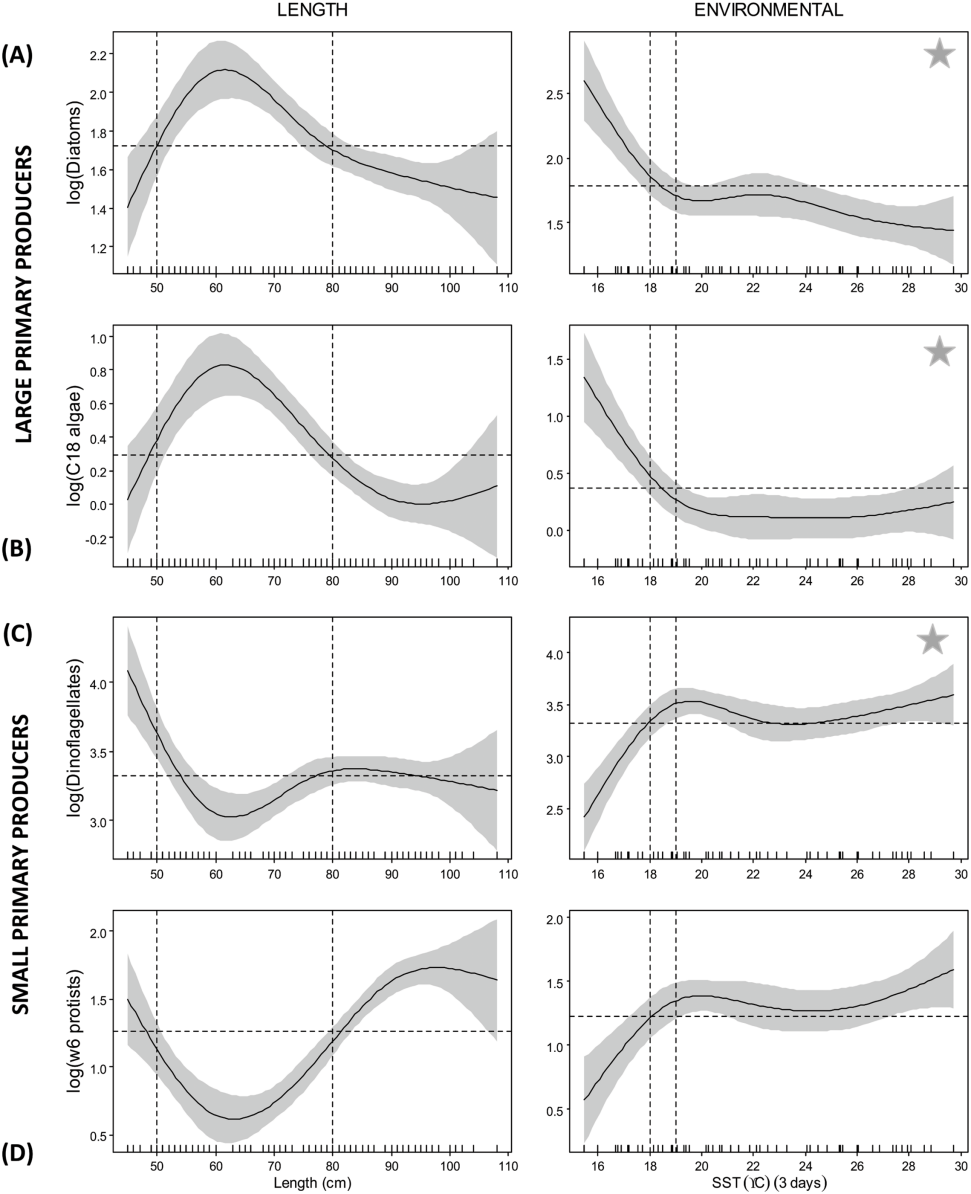
Smoother plots of GAMM predicted albacore fatty acid tracers of large primary producers: (A) diatoms (EPA +14:0), (B) C_18_ algae, (C) dinoflagellates (DHA), and (D) ɷ6 protists. The dashed horizontal black lines represent the intercept in each plot. The dashed vertical lines approximately delineate the range of the explanatory variable above the zero line used as thresholds. The solid grey area bracketing the response curves show the confidence limits of the model and are twice the standard error. A star represents the model fit for that particular response variable with the highest % deviance explained (S3 Table).

**Fig 3:**
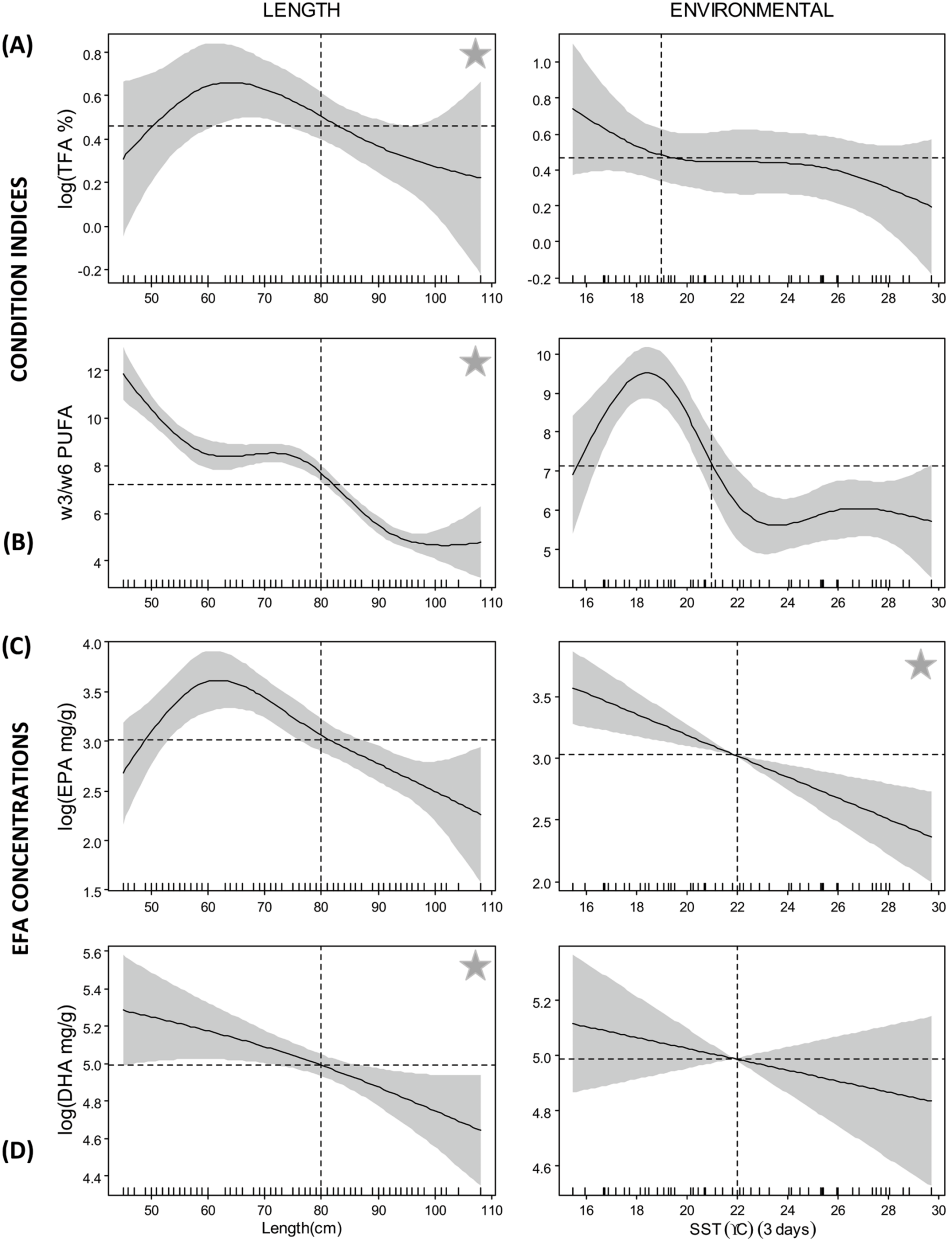
Smoother plots of GAMM predicted albacore condition indicies. (A) total fatty acids (TFA, %) and (B) ratios of ɷ3/ɷ6, and absolute concentrations of the essential fatty acids (EFA): (C) EPA and (D) DHA (mg/100 g of muscle tissue). Black dots are the 29 sampling locations and the lines are contour means. A star represents the best model fit for that particular predictor variable based on % deviance explained (S3 Table). The dashed horizontal black lines represent the intercept in each plot. The dashed vertical lines approximately delineate the range of the explanatory variable above the zero line used as thresholds. The solid grey area bracketing the response curves show the confidence limits of the model; twice the standard error.

Sea surface temperature 3-day composite (SST_3_), as a single explanatory variable, was more important than fork length for the FAT of larger primary producers (diatoms and C_18_ algae) accounting for 61 and 73% of the variability respectively, compared to 60 and 71% for fork length (data in S3 Table). In contrast, for FAT of smaller dinoflagellates, albacore fork length was shown to be more important than SST_3_, particularly for ɷ6 protists. For all SST_3_—FAT models, there was a clear transition at 18–19°C with steepest inclines observed at <18°C (Fig 2). FAT of larger primary producers were more prevalent at lower temperatures than smaller producers. For SST_3_ models of NCI, predicted proportions of TFA and ratios of ɷ3/ɷ6 were highest in cooler waters <19°C and <21°C, respectively (Fig 3A and 3B). Concentrations of EFA also showed similar trends with EPA and DHA decreasing linearly with SST_3_ and the intercept crossing at 22°C (Fig 3C and 3D).

Under a scenario of a 1°C increase in SST_3_, our models predict that in temperate waters (<18°C) FAT of diatoms and C_18_ algae will show an average decrease of 12% and 32% respectively, while those of dinoflagellates and ɷ6 protists will increase 12% and 22%, respectively. In sub-tropical waters (>19°C) a similar increase in SST will only cause a decrease of 4% and <1% for FAT of diatoms and C_18_ algae, respectively, and a mean increase of 0.8% and 1.5% for FAT of dinoflagellates and ɷ6 protists, respectively. Under the same scenario, TFA in albacore would be reduced on average by 12% and 4% in temperate and sub-tropical waters, respectively. In contrast, ratios of ɷ3/ɷ6 will increase on average by 10% in temperate waters and decrease by 3% in sub-tropical waters. Model projections show that if SST increased by 1°C EPA and DHA concentrations in albacore tuna in all waters would decrease by 3% (~8 mg/g) and 1.5% (~16 mg/g), respectively.

Fish size had a clear influence on all predictive variables (FAT, NCI and EFA) with two distinct transitions observed at 50 cm and 80 cm (Figs 2 and 3). For FAT of larger primary producers, lowest relative proportions were observed in albacore <50 cm FL and >80 cm FL, while the opposite trend was found for FAT of smaller primary producers (Fig 2). The best model fit for TFA and ratios of ɷ3/ɷ6 PUFA included length, which alone explained 69 and 60% of the predicted variance, respectively (data in S3 Table). For all NCI and EFA there was a clear transition at 80 cm, with lower proportions of TFA, ratios of ɷ3/ɷ6 and concentrations of EPA and DHA observed in albacore > 80 cm FL (Fig 3).

**Fig 4:**
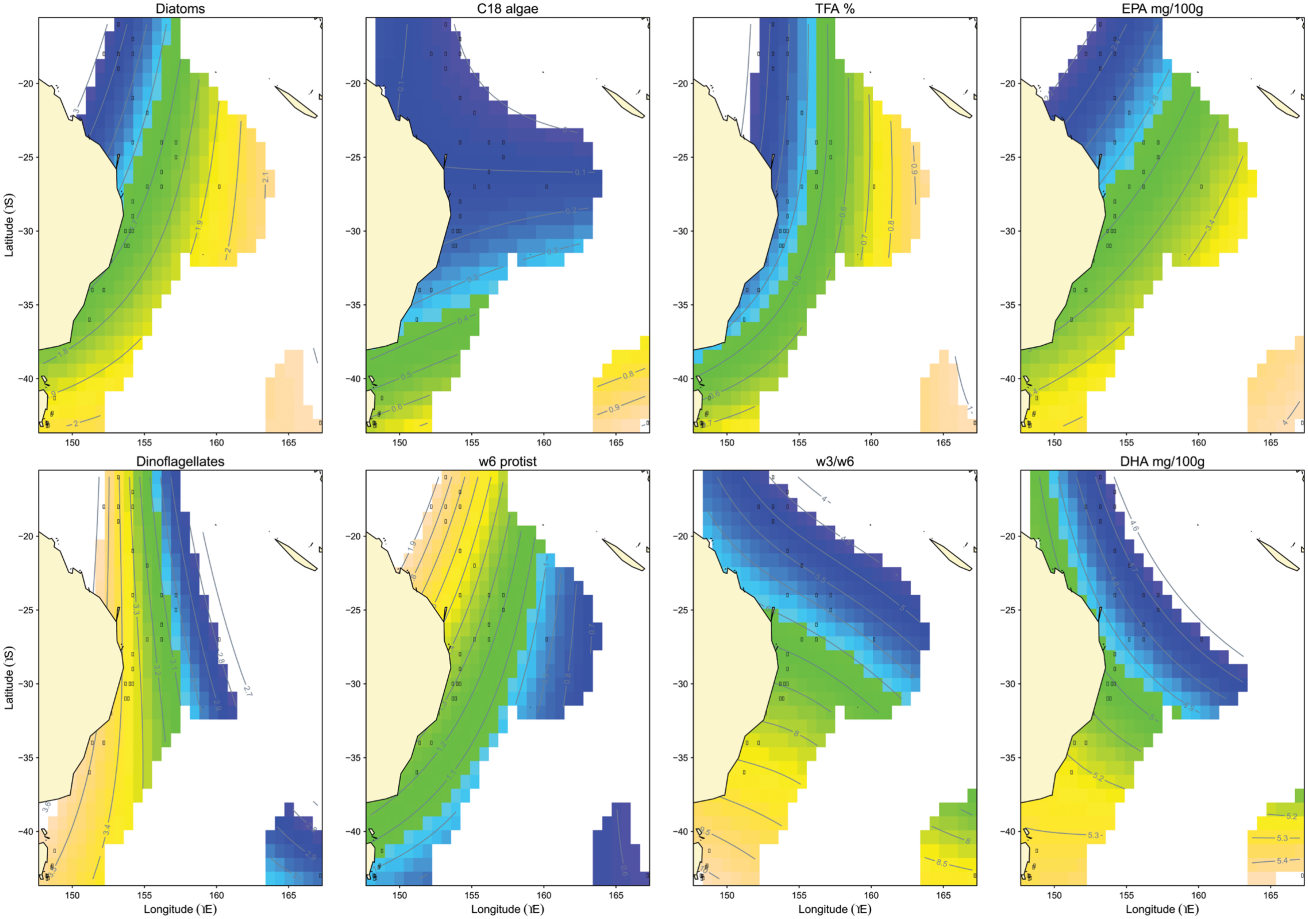
FATscapes or oceanographic contour spatial maps of fatty acid parameters measured in albacore. Black dots represent the 29 sampling locations (Data in S2 Table) and the lines are contour means.

FATscapes were produced for each FAT, NCI, and EFA in albacore tuna muscle and distinct biogeographical zones and latitudinal and longitudinal gradients were displayed (Fig 4). FAT of larger phytoplankton were generally highest in the Tasman Sea with diatoms increasing from north-west to south and C_18_ algae increasing from north to south. In contrast, FAT of smaller phytoplankton was typically highest in the western areas of the Coral Sea with dinoflagellates increasing from north-east to west and ɷ6 protists increasing from south-east to north-west. Clear regional differences between fatty acid parameters of albacore sampled from the east and west Tasman Sea were evident for FAT of dinoflagellates and ɷ6 protists. NCI were highest in the Tasman Sea with TFA increasing from north-west to south-east and ratios of ɷ3/ɷ6 increasing from north-east to south-west. EFA followed similar patterns to their corresponding primary producers with EPA increasing from north-west to south-east, similar to FAT of diatoms and DHA increasing from north-east to south, somewhat similar to FAT of dinoflagellates.

The best model for each FAT was refitted using an independent fatty acid dataset for 30 albacore previously sampled in the Coral and Tasman Sea [21]. For all models, good fits with high degrees of confidence were observed (S1 Fig). High linear correlations (R^2^>0.85) were observed for model predicted variables and our own observations, demonstrating that the models performed well, especially at mean predicted values which were only 2–12% off the observed values.

## Discussion

### Tuna EFA’s predicted to decline with increasing SST

Relative proportions and absolute values of fatty acids in albacore tuna muscle were shown to be highly responsive to variations in SST, particularly in temperate waters < 18°C. Large declines in projected TFA content, FAT of large primary producers and concentrations of EPA and DHA in albacore tuna muscle occurred as a results of a 1°C change in SST. Temperature has a major and direct effect on biochemical and enzymatic (or metabolic) processes, including the synthesis of fatty acids, particularly PUFAs, by primary producers [41, 42] and fish [43]. Controlled feeding studies have shown that at lower temperature treatments the production of saturated and monounsaturated fatty acids decreases while LC-PUFA increase [44–46]. In certain diatom species, TFA and EPA yields have been shown to increase by 3 to 5% when temperature decreased from 25 to 10°C for just 12 hours [47]. Our results demonstrate that temperature driven changes in the fatty acid composition of the phytoplankton community can propagate up the food chain to higher order consumers.

As lipids have a higher calorific value than protein and carbohydrates, a reduction in relative and absolute amounts of TFA content will affect the availability of energy to consumers, and thus ecosystem productivity. In temperate waters, we predicted that EPA and DHA concentrations would decline by 7 and 15 mg/100 g albacore tuna muscle with increasing SST. In marine organisms, LC-PUFA play a critical role in the maintenance of membrane fluidity [48] and in early life-history development [6] meaning that sufficient declines could adversely affect the functional performance and productivity of albacore tuna and their main consumers. For humans, the daily recommended intake of EPA + DHA is 0.25–2 g/d [49, 50] and a resulting omega 3 index (O3I) of greater than 8% (of total fatty acids) is desirable. A reduction in these EFA and the O3I in human nutrition has been linked to increased risk of nutritional related diseases and conditions including coronary heart disease, Alzheimers and multiple sclerosis [51, 52]. Our findings build upon recent concern and evidence that the supply of essential fatty acids (EPA and DHA), particularly from fish harvested from cooler waters, will be insufficient for the projected increase in global human populations [53–55].

Projected changes in all fatty acid parameters were reflected in size-related changes of primary producers. This was particularly the case for FAT of large-celled producers (diatoms, C_18_ algae) and small-celled producers (dinoflagellates and ɷ6 protists) confirming their correct utilization in trophic studies. This result adds another dimension to recent worldwide concerns of climate driven shifts in community composition, abundance and size-structure of primary producers [56–58]. Of particular concern is a global trend towards smaller picophytoplankton (typically <2 μm, such as dinoflagellates) outcompeting larger phytoplankton (such as diatoms) as ocean temperatures and coastal eutrophication increase [59]. Such size-structured changes are projected to decrease the energy transfer efficiencies of marine food chains [14] and EFA are increasingly being recognized as an important contributor. Muller-Navarra et al. [60] assessed the aquatic and fatty acid literature and recognized that phytoplankton-derived EFA (particularly EPA and DHA) are likely to determine energetic transfer efficiency across the plant—animal interface (especially zooplankton biomass), secondary production and the strength of trophic coupling in pelagic food webs. Low transfer efficiencies between primary producers and consumers during cyanobacteria bloom conditions were related to low relative EPA content of the primary producer community [60]. Penhar et al. [17] tested the essential PUFA limitation hypothesis using a deterministic model and showed that essential PUFA can limit zooplankton growth at similar rates to macronutrients (N, P, Si). This agrees with an increasing number of empirical studies (e.g., [61]), and is further supported by captive feeding trials [62].

In addition to temperature, projected values of fatty acid parameters were shown to be influenced by individual fish length of albacore tuna. Ontogenetic changes and spatial variability in fatty acid parameters in albacore tuna are likely related to: (i) known ontogenetic changes in diet [63], (ii) younger organisms having faster metabolic rates, (iii) the fact that smaller albacore spend more time in temperate waters and show reduced annual migration than adults [64], and (iv) dynamic trade-off energy constraints between growth and reproduction [65]. The last point could be significant given that albacore undergo long spawning migrations and that fatty acids are the major metabolic energy resource for fish reproduction [6]. The unusual Gaussian-like curve found in this study was also detected in a concurrent study using stable isotopes [22] and has been detected in dietary studies of albacore in other ocean basins [66]. Using the models developed, most EFA and NCI can be estimated based solely on an animal’s fork length with reasonably high confidence (DE >50%). Smaller albacore caught from temperate waters were observed as having the best nutritional value with highest NCI and EFA concentrations. Such nutritional information can be used to inform consumers where products fit into national food standards and healthy eating guidelines.

### A novel approach of using signature fatty acids and predictive models

The framework presented here includes the development and first time demonstration of FATscapes and, to the authors’ knowledge, the first utilization of GAMMs for organic compounds. Application of FATscapes provides a new approach for describing the past spatial and temporal distributions of phytoplankton produced and essential fatty acids, in addition to being used as a tool for exploring potential impacts of future climate change. This is a significant advancement to previous approaches (such as, PCA, MDS, ANOSIM and PERMANOVA) used to explain variation in fatty acid profiles and trophic markers which are typically based on symmetric matrix of dissimilarities, distances, or their ranks, to test among *a priori* groups based on their fatty acid composition. These previously used and widely applied statistical approaches give valuable information, although they have limited predictive capacity. There is an increasing need to understand changes in trophic relationships over time and space (in the context of climate change and increasing human pressure), and therefore it is vital to use statistical tools that are both exploratory and predictive. GAMMs have the advantage of allowing highly flexible nonparametric relationships between predictor and response variables which are often desirable when there is no *a priori* reason for choosing a response function.

Spatial contour or landscape maps are increasingly produced for naturally occurring isotopes (isoscapes; [40]) to quantify and understand spatio-temporal distributions of isotopic variations in natural systems at beyond regional scales (i.e. at landscape, ocean basin and global scales). They are particularly powerful when attempting to understand macro-ecological patterns responsible for intra-specific and inter-specific differences observed. In a concurrent study, Pethybridge et al. [22] produced isoscapes for bulk carbon δ^13^C and nitrogen δ^15^N isotopes of albacore tuna (including the same individuals analyzed in this study) and then used them to classify distinct bioregions that corresponded to known physical and biological processes. Clear bioregions were also identified from the FATscapes produced in the present study (Fig 4). Predictive values of FAT of large primary producers, NCI, and EFA were all highest in the Tasman Sea and lowest in the Coral Sea reflecting the influence of the East Australian Current and the Tasman Front which extends to ~35°S [67] on diatom/flagellate partitioning [68, 69]. Predicted FAT of smaller producers were highest in the Coral sea and eastern coast of Australia. Longitudinal gradients reflect distinct inshore/offshore communities of dominant phytoplankton [41, 70].

### Caveats and future research

The utility of primary producer FATs as primary drivers contributing to lipid profiles of tertiary consumers is complicated by mid-trophic level dynamics and unknown trophic transfer efficiencies of fatty acid nutrients from lower to higher trophic groups. Furthermore, the migratory nature of albacore tunas could drastically affect the spatial resolution of our results. This study attempted to reduce this error by including composite environmental data just (3 days) before and moderately (15 and 30 days) before tuna were sampled. Surprisingly, however, all predictive variables responded better to the shorter-term (3 day composite) predictive variables, rather than longer-term data that encompass a period of time when fatty acids of muscle tissue are replaced by those more representative of their diet (with turnover rates of muscle being ~3–10 weeks). Future studies should consider a greater time lapse (e.g. 30–90 days) between the date top consumers were sampled and when environmental data are acquired. Future work should also aim to combine data on the biochemical composition of individual fish and their movement patterns (through tagging studies) so that better relationships between diet and location (degree of residency) can be made.

It is important to note that the FATscapes produced in this study represent the spatial distribution of fatty acids of albacore tuna muscle tissue sampled over a two year period and could reflect differences for a given season, particularly in the temperate areas of the study where there is greater seasonality of the prevailing physical properties and phytoplankton dynamics. As fatty acids in tuna muscle tissue have faster turnover rates, compared to stable isotopes (~2–4 months, [71]), they are considered to be better tracers to investigate finer scale time-series changes in regional food webs and may provide an earlier warning to fishery managers for potential shifts. Whilst time of the year (DOY; models 6 and 7) did not appear in any of the best models, we recommend that future research look to acquire fatty acid data for samples collected over a finer temporal scale so that more detailed (e.g. seasonal-based) FATscapes are produced. Likewise, finer-scale spatial predictive maps of fatty acids could be achieved through the collection and analysis of producers or consumers with smaller movement patterns.

## Conclusions

We developed a framework that can quantify and predict the spatial and temporal variability of various fatty acid parameters measured in the tissues of large commercially important marine predators. Using this framework we found that the supply and availability of EFA are likely to be negatively affected by increases in sea surface temperature, particularly in temperate environments. The changes in fatty acid proportions and concentrations reflect shifts in phytoplankton dynamics, including species composition and size structure, and describe mechanisms by which marine energy pathways and ecosystem stability could change. The models presented can be used to infer the fatty acid composition of other generalist marine top predators with similar movement patterns and metabolic parameters. They can also be used to understand potential future impacts driven by natural and anthropogenic climate change. Indicators of climate change are thought to be important in planning adaptation strategies for harvested marine species [72]. Indeed, measuring the fatty acid composition and content of regularly fished resources offers a cost effective approach to understand trophic structure dynamics and their response to change. International efforts are now needed to assess broad, global-scale patterns in marine fatty acids similar to that being undertaken for isotopes [73] and trace elements [74].

Assessing and monitoring entire ecosystem dynamics from nutrient inputs to top predators is notoriously difficult, largely because there are very few methods that can detect multi-scale and food web integrated signals [75]. As fatty acids are derived from primary producers and are essential for marine organisms to grow, reproduce and survive, they provide excellent tools to simultaneously examine bottom-up drivers of energy transfer, trophic structure and nutritional condition or health at the organismal and community scales.

## Supporting Information

S1 Fig Model cross-validation of fatty acid parameters in albacore. Best model fit (black line) is plotted against observations (black points) from an independent data set (Parrish et al. [21]).
(EPS)

S1 Table Results (mean ± standard deviation) of biological and fatty acid data for albacore collected from different FATscape bioregions in the SW Pacific Ocean. N—Sample sizes, FL—fork length, SST3 –sea surface temperature 3 day composite, Chla8 –chlorophyll-a concentrations of a 8-day composite, ɷ –omega, TFA—total fatty acids, SFA—saturated fatty acids, MUFA—monounsaturated fatty acids, PUFA—polyunsaturated fatty acids, iso-FA—iso-methyl branched fatty acids. Fatty acid data is presented as mean area percent of total fatty acids.
(DOCX)

S2 Table Collection, biological, environmental and biochemical data used in this study. (CSV)

S3 Table Comparable performance of 13 models tested for each fatty acid parameter measured in albacore muscle. Fatty acid parameters included: fatty acid tracer (FAT), nutritional condition indices (NCI) and essential fatty acid (DHA and EPA) concentrations. K—number of parameters estimated in the model, AICc and ΔAICc—Akaike information criteria for small sample sizes and relative changes, LL—model log likelihood, %DE—percent deviance explained. The best model fit is in bold.
(DOCX)

## References

[1] Das UN (2006) Essential fatty acids-a review. Current pharmaceutical biotechnology 7(6): 467–482. 17168664 10.2174/138920106779116856

[2] Paulsen M, Clemmesen C, Malzahn AM (2014) Essential fatty acid (docosahexaenoic acid, DHA) availability affects growth of larval herring in the field. Mar Biol 161(1): 239–244.

[3] Cunnane SC, Crawford MA (2014) Energetic and nutritional constraints on infant brain development: Implications for brain expansion during human evolution. J Hum Evol 77, 88–98. 10.1016/j.jhevol.2014.05.001 24928072

[4] Tocher DR (2003) Metabolism and functions of lipids and fatty acids in teleost fish. Rev Fish Sci 11:107–184.

[5] Nakamura MT, Cho HP, Xu J, Tang Z, Clarke SD (2001) Metabolism and function of highly unsaturated fatty acids: an update. Lipids 36(9): 961–964. 11724468 10.1007/s11745-001-0806-5

[6] Sargent JR, Tocher DR, Bell JG (2002) The lipids. Fish Nutr 3:181–257.

[7] Kiessling A, Pickova J, Johansson L, Åsgard T, Store-bakken T, Kiessling K-H (2001) Changes in fatty acid composition in muscle and adipose tissue of farmed rainbow trout (*Oncorhynchus mykiss*) in relation to ration and age. Food Chem 73: 271–284.

[8] Tocher DR (2010) Fatty acid requirements in ontogeny of marine and freshwater fish. Aquac Res 41(5): 717–732.

[9] Okuyama H, Orikasa Y, Nishida T, Watanabe K, Morita N (2007) Bacterial genes responsible for the biosynthesis of eicosapentaenoic and docosahexaenoic acids and their heterologous expression. Appl Environ Microbiol 73: 665–670. 17122401 10.1128/AEM.02270-06 PMC1800774

[10] Parrish CC (2013) Lipids in marine ecosystems. ISRN Oceanogr 2013:1–16.

[11] Dalsgaard J, St John M, Kattner G, Müller-Navarra D, Hagen W (2003) Fatty acid trophic markers in the pelagic marine environment. Adv Mar Biol 46: 225–340. 14601414 10.1016/s0065-2881(03)46005-7

[12] Sommer U, Lengfellner K (2008) Climate change and the timing, magnitude, and composition of the phytoplankton spring bloom. Global Change Biol 14(6): 1199–1208.

[13] Klais R, Tamminen T, Kremp A, Spilling K, Olli K (2011) Decadal-scale changes of dinoflagellates and diatoms in the anomalous Baltic Sea spring bloom. PLOS ONE 6(6): e21567 10.1371/journal.pone.0021567 21747911 PMC3126836

[14] Polovina JJ, Woodworth PA (2012) Declines in phytoplankton cell size in the subtropical oceans estimated from satellite remotely-sensed temperature and chlorophyll, 1998–2007. Deep Sea Res II 77: 82–88.

[15] Polovina JJ, Dunne JP, Woodworth PA, Howell EA (2011) Projected expansion of the subtropical biome and contraction of the temperate and equatorial upwelling biomes in the North Pacific under global warming. ICES J Mar Sci 10.1093/icesjms/fsq198

[16] Gladyshev MI, Sushchik NN, Anishchenko OV, Makhutova ON, Kolmakov VI, Kalachova GS, et al (2011) Efficiency of transfer of essential polyunsaturated fatty acids versus organic carbon from producers to consumers in a eutrophic reservoir. Oecologia 165(2): 521–531. 10.1007/s00442-010-1843-6 21107868

[17] Perhar G, Arhonditsis GB, Brett MT (2012) Modelling the role of highly unsaturated fatty acids in planktonic food web processes: a mechanistic approach. Env Rev 20(3): 155–172.

[18] Reuss N, Poulsen L (2002) Evaluation of fatty acids as biomarkers for a natural plankton community. A field study of a spring bloom and a post-bloom period off West Greenland. Mar Biol 141(3): 423–434.

[19] Rossi S, Sabatés A, Latasa M, Reyes E (2006) Lipid biomarkers and trophic linkages between phytoplankton, zooplankton and anchovy (*Engraulis encrasicolus*) larvae in the NW Mediterranean. J Plankton Res 28(6): 551–562.

[20] Pethybridge H, Bodin N, Arsenault-Pernet EJ, Bourdeix JH, Brisset B, Bigot JL, et al (2014) Temporal and inter-specific variations in forage fish feeding conditions in the NW Mediterranean: lipid content and fatty acid compositional changes. Mar Ecol Prog Ser 512:39–54.

[21] Parrish CC, Pethybridge H, Young J, Nichols PD (2015) Spatial variation in fatty acid trophic markers in albacore tuna from the south western Pacific Ocean—A potential ‘tropicalization’ signal. Deep Sea Res 113: 199–207.

[22] Pethybridge HP, Young JW, Kuhnert PM, Farley JH (2015) Stable isotopes of albacore tuna show bioregions and a potential regime shift in the south-western Pacific Ocean. Prog Oceanogr In press.

[23] Ridgeway KR, Hill K (2012) East Australian Current, In Marine Climate Change Impacts and Adaptation Report Card for Australia 2012, eds Poloczanska ES, Hobday AJ, Richardson AJ (CSIRO Climate Adaptation Flagship, Hobart), pp 47–59. ISBN 978-0-643-10928-5

[24] Wu L, Cai W, Zhang L, Nakamura H, Timmermann A, Joyce T, Joyce T, et al (2012) Enhanced warming over the global subtropical western boundary currents. Nature Climate Change 2: 161–166.

[25] Thompson PA, Baird ME, Ingleton T, Doblin MA (2009) Long-term changes in temperate Australian coastal waters: implications for phytoplankton. Mar Ecol Prog Ser 394:1–19.

[26] Hobday AJ, Pecl GT (2014) Identification of global marine hotspots: sentinels for change and vanguards for adaptation action. Rev Fish Biol Fisher 24(2): 415–425.

[27] Hartog J, Hobday AJ (2011) SDODE: Spatial dynamics ocean data explorer User Guide v3. CSIRO Marine and Atmospheric Research, Hobart.

[28] Barnes C, Irigoien X, De Oliveira JA, Maxwell D, Jennings S (2011) Predicting marine phytoplankton community size structure from empirical relationships with remotely sensed variables. J Plankton Res 33(1): 13–24.

[29] Parrish CC, Nichols PD, Pethybridge H, Young J (2015) Direct determination of fatty acids in fish tissue in order to define and then quantify top predator trophic connections in the marine environment. Oecologia 177(1), 85–95. 10.1007/s00442-014-3131-3 25376156

[30] Parrish CC, French VM, Whiticar MJ (2012) Lipid class and fatty acid composition of copepods (*Calanus finmarchicus*, *C*. *glacialis*, *Pseudocalanus* sp., *Tisbe furcata* and *Nitokra lacustris*) fed various combinations of autotrophic and heterotrophic protists. J Plankton Res 34(5): 356–375.

[31] Viso AC, Marty JC (1993) Fatty acids from 28 marine microalgae. Phytochem 34(6): 1521–1533.

[32] Sargent JR, Parkes RJ, Mueller-Harvey I, Henderson RJ (1987) Lipid biomarkers in marine ecology In: Sleigh MA, editor. Microbes in the Sea. Chichester: Ellis Horwood Ltd pp. 119–138.

[33] Parrish CC (2009) Essential fatty acids in aquatic food webs In: Arts MT, Brett MT, Kainz M, editors. Lipids in Aquatic Ecosystems. New York: Springer pp. 309–326.

[34] Metz JG, Roessler P, Facciotti D, Levering C, Dittrich F, Lassner M, et al (2001) Production of polyunsaturated fatty acids by polyketide synthase in both prokaryotes and eukaryotes. Science 293:290–293 11452122 10.1126/science.1059593

[35] Loef M, Walach H (2013) The omega-6/omega-3 ratio and dementia or cognitive decline: a systematic review on human studies and biological evidence. J Nutr Gerontol Geriatr 32, 1–23. 10.1080/21551197.2012.752335 23451843

[36] Wood S (2006) Generalized additive models: an introduction with R. Bocca Raton: Chapman & Hall/CRC.

[37] Burnham KP, Anderson DR (2004) Multimodel inference understanding AIC and BIC in model selection. Sociol method Res 33(2): 261–304.

[38] Wood S (2011) gamm4: Generalized additive mixed models using mgcv and lme4. R package version 0.1–2.

[39] R Development Core Team (2011) R: A language and environment for statistical computing, reference index version 2. R Foundation for Statistical Computing, Vienna, Austria. ISBN 3-900051-07-0

[40] West JB, Bowen GJ, Dawson TE, Tu KP (2010) Understanding movement, pattern, and process on Earth through isotope mapping. New York: Springer.

[41] Thompson PA, Guo MX, Harrison PJ (1992) Effects of variation in temperature. I. On the biochemical composition of eight species of marine phytoplankton. J Phycol 28:481–488.

[42] Renaud S, Zhou H, Parry D, Thinh LV, Woo K (1995) Effect of temperature on the growth, total lipid content and fatty acid composition of recently isolated tropical microalgae *Isochrysis* sp., *Nitzschia closterium*, *Nitzschia paleacea*, and commercial species *Isochrysis* sp.(clone T. ISO). J Appl Phycol 7:595–602.

[43] Tocher DR, Sargent JR (1990) Effect of temperature on the incorporation into phospholipid classes and metabolism via desaturation and elongation of n-3 and n-6 polyunsaturated fatty acids in fish cells in culture. Lipids 25:435–442. 10.1111/j.1471-4159.1990.tb04918.x 2338561

[44] Scholz B, Liebezeit G (2013) Compatible solutes and fatty acid composition of five marine intertidal microphytobenthic Wadden Sea diatoms exposed to different temperature regimes. Diatom Res 28: 337–358.

[45] Harwood JL (1988) Fatty acid metabolism. Ann Rev Plant Physiol Plant Mol Biol 39:101–138.

[46] Renaud SM, Thinh LV, Lambrinidis G, Parry DL (2002) Effect of temperature on growth, chemical composition and fatty acid composition of tropical Australian microalgae grown in batch cultures. Aquaculture 211(1): 195–214.

[47] Jiang H, Gao K (2004) Effects of lowering temperature during culture on the production of polyunsaturated Fatty Acids in the Marine Diatom *Phaeodactylum tricornutum* (Bacillariophyceae). J Phycol 40:651–654.

[48] Arts MT, Kohler CC (2009) Health and condition in fish: the influence of lipids on membrane competency and immune response In: Arts MT, Brett MT, Kainz M, editors. Lipids in Aquatic Ecosystems. New York: Springer pp 237–256.

[49] FAO-WHO (2010) Fats and Fatty Acids in Human Nutrition. Rome: FAO Food and nutrition paper 91, Report of an expert consultation, Geneva, November 10–14, 2008. 21812367

[50] Aranceta J, Pérez-Rodrigo C (2012) Recommended dietary reference intakes, nutritional goals and dietary guidelines for fat and fatty acids: a systematic review. British J Nutr 107(S2): S8–S22. 10.1017/S0007114512001444 22591906

[51] Wijendran V, Hayes KC (2004) Dietary n-6 and n-3 fatty acid balance and cardiovascular health. Annu Rev Nutr 24: 597–615. 15189133 10.1146/annurev.nutr.24.012003.132106

[52] Uauy R, Dangour AD (2006) Nutrition in brain development and aging: role of essential fatty acids. Nutr Rev 64(s2): S24–S33. 16770950 10.1301/nr.2006.may.s24-s33

[53] Deckelbaum RJ, Torrejon C (2012) The omega-3 fatty acid nutritional landscape: health benefits and sources. J Nutr 142(3): 587S–591. 10.3945/jn.111.148080 22323763 PMC3278270

[54] Naylor RL, Hardy RW, Bureau DP, Chiu A, Elliott M, Farrell AP, et al (2009) Feeding aquaculture in an era of finite resources. Proc Natl Acad Sci 106(36): 15103–15110. 10.1073/pnas.0905235106 19805247 PMC2741212

[55] Budge SM, Devred E, Forget MH, Stuart V, Trzcinski MK, Sathyendranath S, et al (2014) Estimating concentrations of essential omega-3 fatty acids in the ocean: supply and demand. ICES J Mar Sci

[56] Boyd PW, Strzepek R, Fu F, Hutchins DA (2010) Environmental control of open-ocean phytoplankton groups: Now and in the future. Limnology and Oceanogr 55(3): 1353.

[57] Alvain S, Le Quéré C, Bopp L, Racault MF, Beaugrand G, Dessailly D, et al (2013) Rapid climatic driven shifts of diatoms at high latitudes. Remote Sens Envir 132: 195–201.

[58] Lewandowska AM, Boyce DG, Hofmann M, Matthiessen B, Sommer U, Worm B (2014) Effects of sea surface warming on marine plankton. Ecol Letters 17(5): 614–623. 10.1111/ele.12265 24575918

[59] Suikkanen S, Pulina S, Engström-Öst J, Lehtiniemi M, Lehtinen S, Brutemark A (2013) Climate change and eutrophication induced shifts in northern summer plankton communities. PLOS ONE 8(6): e66475 10.1371/journal.pone.0066475 23776676 PMC3680480

[60] Müller-Navarra DC, Brett MT, Liston AM, Goldman CR (2000) A highly unsaturated fatty acid predicts carbon transfer between primary producers and consumers. Nature 403:74–77. 10638754 10.1038/47469

[61] Park S, Brett MT, Muller-Navarra DC, Goldman CR (2002) Essential fatty acid content and the phosphorus to carbon ratio in cultured algae as indicators of food quality for Daphnia. Freshwater Biol 47(8), 1377–1390.

[62] Glencross BD (2009) Exploring the nutritional demand for essential fatty acids by aquaculture species. Revs Aquac 1:71–124.

[63] Young JW, Bradford R, Lamb TD, Clementson LA, Kloser R, Galea H (2001) Yellowfin tuna (*Thunnus albacares*) aggregations along the shelf break off south-eastern Australia: links between inshore and offshore processes. Mar Freshwat Res 52(4): 463–474.

[64] Farley JH, Williams AJ, Hoyle SD, Davies CR, Nicol SJ (2013) Reproductive dynamics and potential annual fecundity of South Pacific albacore tuna (*Thunnus alalunga*). *PLOS ONE* 8(4):e60577. 23565258 10.1371/journal.pone.0060577 PMC3614989

[65] Claramunt G, Serra R, L Castro L, Cubillos L (2007) Is the spawning frequency dependent on female size? Empirical evidence in *Sardinops sagax* and *Engraulis ringens* off northern Chile. Fish Res 85: 248–257.

[66] Goñi N, Logan J, Arrizabalaga H, Jarry M, Lutcavage M (2011) Variability of albacore (*Thunnus alalunga*) diet in the Northeast Atlantic and Mediterranean Sea. Mar Biol 158(5): 1057–1073.

[67] Ridgway K R, Dunn JR (2003) Mesoscale structure of the mean East Australian Current System and its relationship with topography. Prog Oceanogr 56(2): 189–222.

[68] Ajani P, Lee R, Pritchard T, Krogh M (2001) Phytoplankton dynamics at a long-term coastal station off Sydney, Australia. J Coastal Res 34:60–73.

[69] Jeffrey SW, Hallegraeff GM (1990) Phytoplankton ecology of Australasian waters In: Clayton MN, King RJ, editors. Biology of Marine Plants. Melbourne: Longman Cheshire pp 310–348.

[70] Bax N J, Burford M, Clementson L, Davenport S (2001) Phytoplankton blooms and production sources on the south-east Australian continental shelf. Mar Freshwater Res 52(4): 451–462.

[71] Logan JM, Jardine TD, Miller TJ, Bunn SE, Cunjak RA, Lutcavage ME (2008) Lipid corrections in carbon and nitrogen stable isotope analyses: comparison of chemical extraction and modelling methods. J Animal Ecol 77(4): 838–846. 10.1111/j.1365-2656.2008.01394.x 18489570

[72] Hobday AJ (2010) Ensemble analysis of the future distribution of large pelagic fishes off Australia. Prog Oceanogr 86(1): 291–301.

[73] Young JW, Olson RJ, Ménard F, Kuhnert PM, Duffy LM, Allain V, et al (2015) Setting the stage for a global-scale trophic analysis of marine top predators: a multi-workshop review. *Rev* Fish Biol Fisher 25(1): 261–272.

[74] McMahon KW, Hamady LL, Thorrold SR (2013) A review of ecogeochemistry approaches to estimating movements of marine animals. Limnol Oceanogr 58(2):697714.

[75] Painting SJ, Van der Molen J, Parker ER, Coughlan C, Birchenough S, Bolam S, et al (2013) Development of indicators of ecosystem functioning in a temperate shelf sea: a combined fieldwork and modelling approach. Biogeochem 113(1–3): 237–257.

