# Supplementary material for: Spatial Patterns and Temperature Predictions of Tuna Fatty Acids: Tracing Essential Nutrients and Changes in Primary Producers
Source: PLoS One. 2015 Jul 2;10(7):e0131598. doi: 10.1371/journal.pone.0131598 (PMC4489677; doi:10.1371/journal.pone.0131598)
Supplement: S1 Table — N—Sample sizes, FL—fork length, SST3 –sea surface temperature 3 day composite, Chla8 –chlorophyll-a concentrations of a 8-day composite, ɷ –omega, TFA—total fatty acids, SFA—saturated fatty acids, MUFA—monounsaturated fatty acids, PUFA—polyunsaturated fatty acids, iso-FA—iso-methyl branched fatty acids. Fatty acid data is presented as mean area percent of total fatty acids. (DOCX) [file pone.0131598.s002.docx]

**SUPPORTING INFORMATION (SI)**

**S1 Table.** Sample sizes (N) and mean ± SD results of biological and major fatty acid data (as % of total fatty acids) for albacore tuna muscle tissue collected in different FATscape bioregions in 2009 and 2010 in south-west Pacific Ocean. FL – fork length, SST_3_ – sea surface temperature 3 day composite, Chl*a*_8_ – chlorophyll-*a* concentrations of a 8-day composite, ω – omega, TFA – total fatty acids, SFA – saturated fatty acids, MUFA – monounsaturated fatty acids, PUFA – polyunsaturated fatty acids, iso-FA – iso-methyl branched fatty acids.

|  | Tasman Sea - west | | Tasman Sea - NZ | | Central - inshore | | Central -offshore | | Coral Sea - south | | Coral Sea - north | |
| --- | --- | --- | --- | --- | --- | --- | --- | --- | --- | --- | --- | --- |
| N | 16 |  | 31 |  | 42 |  | 17 |  | 31 |  | 17 |  |
| FL (cm) | 55.6 | ±9.3 | 59.3 | ±8.0 | 80.3 | ±8.6 | 92.6 | ±5.4 | 90.9 | ±7.1 | 94.0 | ±4.6 |
| Age (yr) | 1.5 | ±0.6 | 1.8 | ±0.6 | 3.9 | ±1.6 | 4.4 | ±1.3 | 5.2 | ±1.6 | 6.3 | ±2.2 |
| month | 2.4 | ±0.9 | 2.6 | ±1.2 | 6.3 | ±1.06 | 7.0 | ±4.9 | 5.2 | ±0.7 | 6.0 | ±4.4 |
| year | 2009.3 | ±0.5 | 2010.0 | ±0.0 | 2009.5 | ±0.5 | 2009.5 | ±0.5 | 2009.5 | ±0.5 | 2009.4 | ±0.5 |
| Latitude | -42.77 | ±0.51 | -43.00 | ±0.00 | -31.17 | ±1.92 | -26.41 | ±0.71 | -23.12 | ±1.27 | -17.65 | ±0.95 |
| Longitude | 148.21 | ±0.17 | 168.00 | ±0.00 | 152.96 | ±0.92 | 156.53 | ±1.37 | 155.00 | ±1.12 | 153.16 | ±0.64 |
| SST_3_ °C | 17.58 | ±0.82 | 16.36 | ±0.58 | 20.51 | ±1.29 | 25.72 | ±2.62 | 25.15 | ±1.14 | 27.20 | ±1.62 |
| Chl*a*_8_ mg m^-3^ | 0.42 | ±0.14 | 0.37 | ±0.12 | 0.39 | ±0.25 | 0.07 | ±0.06 | 0.14 | ±0.08 | 0.05 | ±0.02 |
| TFA % ww | 2.59 | ±2.09 | 4.14 | ±1.99 | 1.54 | ±0.83 | 2.39 | ±0.93 | 1.10 | ±0.51 | 1.50 | ±0.74 |
| ω3/ω6 PUFA | 10.39 | ±1.52 | 8.08 | ±2.57 | 7.48 | ±1.52 | 7.32± | 1.83 | 5.34 | ±1.41 | 4.84 | ±0.78 |
| 14:0 ^D^ | 1.67 | ±1.39 | 4.19 | ±3.53 | 1.33 | ±0.93 | 1.65 | ±0.87 | 2.12 | ±1.67 | 1.37 | ±0.94 |
| 16:0 | 20.34 | ±1.67 | 22.98 | ±5.78 | 20.71 | ±2.65 | 22.26 | ±2.68 | 25.66 | ±5.91 | 23.36 | ±3.46 |
| 18:0 | 8.98 | ±1.65 | 8.92 | ±1.63 | 9.20 | ±1.29 | 10.43 | ±1.76 | 10.14 | ±3.15 | 9.54 | ±1.48 |
| 16:1ω7 | 2.09 | ±1.47 | 2.65 | ±1.49 | 1.89 | ±1.01 | 2.12 | ±1.04 | 2.48 | ±1.75 | 1.96 | ±1.00 |
| 18:1ω9 | 7.90 | ±3.37 | 10.78 | ±3.57 | 10.85 | ±4.25 | 11.90 | ±4.21 | 10.13 | ±3.92 | 9.64 | ±1.94 |
| 18:1ω7 | 2.62 | ±0.29 | 2.86 | ±0.73 | 2.35 | ±0.43 | 2.66 | ±0.34 | 2.43 | ±0.68 | 2.46 | ±0.48 |
| Σ C_18_ PUFA ^A^ | 1.96 | ±0.76 | 3.49 | ±2.01 | 1.23 | ±0.56 | 1.25 | ±0.48 | 1.11 | ±0.39 | 1.22 | ±0.70 |
| 20:4ω6 ^P^ | 1.69 | ±0.37 | 1.34 | ±0.65 | 2.34 | ±0.96 | 2.19 | ±1.20 | 2.48 | ±1.09 | 3.11 | ±0.82 |
| 20:5ω3 (EPA) ^D^ | 5.79 | ±0.97 | 5.60 | ±1.62 | 4.04 | ±1.10 | 3.91 | ±0.88 | 3.18 | ±0.94 | 3.26 | ±0.86 |
| 22:5ω6 ^P^ | 0.71 | ±0.28 | 0.64 | ±0.38 | 1.31 | ±0.65 | 1.03 | ±0.39 | 1.63 | ±0.89 | 2.33 | ±0.70 |
| 22:6ω3 (DHA) ^F^ | 34.83 | ±8.93 | 24.97 | ±13.70 | 32.09 | ±8.84 | 27.32 | ±8.10 | 24.58 | ±10.13 | 29.85 | ±6.30 |
| **Σ SFA** | **32.75** | **±2.48** | **38.61** | **±9.01** | **33.25** | **±3.81** | **36.49** | **±3.97** | **40.87** | **±9.16** | **36.88** | **±5.54** |
| **Σ MUFA** | **17.62** | **±6.73** | **20.60** | **±6.69** | **20.64** | **±7.23** | **22.64** | **±6.60** | **20.68** | **±6.56** | **18.02** | **±3.75** |
| **Σ PUFA** | **47.86** | **±7.59** | **38.63** | **±13.52** | **43.72** | **±9.47** | **38.40** | **±8.40** | **35.65** | **±11.33** | **42.50** | **±7.39** |
| **Σ iso-FA** | **0.50** | **±0.20** | **0.78** | **±0.43** | **0.55** | **±0.24** | **0.63** | **±0.25** | **0.57** | **±0.23** | **0.50** | **±0.18** |

**Superscripts:** D – diatoms, F – flagellates, P – ω6 protists, A – C_18_ algae.
