# Supplementary material for: Spatial Patterns and Temperature Predictions of Tuna Fatty Acids: Tracing Essential Nutrients and Changes in Primary Producers
Source: PLoS One. 2015 Jul 2;10(7):e0131598. doi: 10.1371/journal.pone.0131598 (PMC4489677; doi:10.1371/journal.pone.0131598)
Supplement: S3 Table — Fatty acid parameters included: fatty acid tracer (FAT), nutritional condition indices (NCI) and essential fatty acid (DHA and EPA) concentrations. K—number of parameters estimated in the model, AICc and ΔAICc—Akaike information criteria for small sample sizes and relative changes, LL—model log likelihood, %DE—percent deviance explained. The best model fit is in bold. (DOCX) [file pone.0131598.s004.docx]

**SUPPORTING INFORMATION (SI)**

**SI3 Table.** Comparable performance of 13 models tested for each fatty acid tracer (FAT), nutritional condition indices (NCI) and essential fatty acid (DHA and EPA) concentrations measured in tuna muscle. K – number of parameters estimated in the model, AICc and ΔAICc – Akaike information criteria for small sample sizes and relative changes, LL – model log likelihood, %DE – percent deviance explained. The best model fit is in bold.

| **Response variable** | # | **Model type** | **K** | **AICc** | **ΔAICc** | **LL** | **%DE** |
| --- | --- | --- | --- | --- | --- | --- | --- |
| **Diatoms** | 1 | *Null* | 3 | 128.69 | 29.71 | -61.26 |  |
| **Log(EPA+14:0)** | 2 | s(FL) | 5 | 115.62 | 16.65 | -52.61 | 60.04 |
|  | 3 | s(SST_3_) | 5 | 104.55 | 5.13 | -46.97 | 61.37 |
|  | 4 | s(lat,long) | 9 | 130.25 | 31.28 | -55.50 | 54.25 |
|  | 5 | s(FL)+s(lat,long) | 11 | 121.81 | 22.84 | -48.98 | 61.96 |
|  | 6 | s(FL)+s(DOY) | 6 | 117.48 | 18.5 | -52.45 | 60.56 |
|  | 7 | s(FL)+s(lat,long)+s(DOY) | 12 | 124.16 | 25.19 | -48.98 | 63.38 |
|  | **8** | **s(FL)+s(SST_3_)** | **7** | **98.43** | **0** | **-42.1** | **66.92** |
|  | 9 | s(FL)+s(SST_15_) | 7 | 101.12 | 1.69 | -43.01 | 65.86 |
|  | 10 | s(FL)+s(Chl*a*_8_) | 7 | 117.28 | 18.31 | -51.26 | 62.09 |
|  | 11 | s(FL)+s(Chl*a*_30_) | 7 | 119.79 | 20.82 | -52.51 | 60.68 |
|  | 12 | s(FL)+s(SST_3_)+S(Chl*a*_8_) | 9 | 104.52 | 5.49 | -42.63 | 66.97 |
|  | 13 | s(FL)+s(M_B50_) | 7 | 114.7 | 15.56 | -49.97 | 63.3 |
| **C_18_ algae** | 1 | *Null* | 3 | 198.78 | 31.21 | -96.31 |  |
| **log(C_18_** | 2 | s(FL) | 5 | 178.15 | 10.58 | -83.87 | 70.72 |
| **PUFA)** | 3 | s(SST_3_) | 5 | 173.70 | 6.13 | -87.11 | 73.01 |
|  | 4 | s(lat,long) | 9 | 205.50 | 37.93 | -93.12 | 64.81 |
|  | 5 | s(FL)+s(lat,long) | 11 | 193.4 | 25.83 | -84.77 | 70.46 |
|  | 6 | s(FL)+s(DOY) | 6 | 180.31 | 12.74 | -83.87 | 70.72 |
|  | 7 | s(FL)+s(lat,long)+s(DOY) | 12 | 195.75 | 28.18 | -84.77 | 70.46 |
|  | **8** | **s(FL)+s(SST_3_)** | **7** | **167.57** | **0** | **-76.4** | **73.61** |
|  | 9 | s(FL)+s(SST_15_) | 7 | 168.35 | 0.78 | -76.79 | 73.64 |
|  | 10 | s(FL)+s(Chl*a*_8_) | 7 | 180.74 | 13.17 | -82.98 | 71.83 |
|  | 11 | s(FL)+s(Chl*a*_30_) | 7 | 182.59 | 15.02 | -83.91 | 70.86 |
|  | 12 | s(FL)+s(SST_3_)+S(Chl*a*_8_) | 9 | 172.67 | 5.1 | -76.71 | 73.68 |
|  | 13 | s(FL)+s(M_B50_) | 7 | 176.37 | 8.8 | -80.8 | 72.71 |
| **Dinoflagellates** | 1 | *Null* | 3 | 190.28 | 23.76 | -92.06 |  |
| **log(DHA)** | 2 | s(FL) | 5 | 181.07 | 14.55 | -85.33 | 36.27 |
|  | 3 | s(SST_3_) | 5 | 178.87 | 12.35 | -87.11 | 32.94 |
|  | 4 | s(lat,long) | 9 | 189.16 | 20.21 | -87.11 | 26.43 |
|  | 5 | s(FL)+s(lat,long) | 11 | 183.46 | 16.94 | -79.8 | 36.11 |
|  | 6 | s(FL)+s(DOY) | 6 | 181.72 | 15.19 | -84.57 | 40.38 |
|  | 7 | s(FL)+s(lat,long)+s(DOY) | 12 | 182.49 | 15.97 | -78.14 | 40.02 |
|  | **8** | **s(FL)+s(SST_3_)** | **7** | **166.52** | **0** | **-75.88** | **42.93** |
|  | 9 | s(FL)+s(SST_15_) | 7 | 170.37 | 3.85 | -77.8 | 42.07 |
|  | 10 | s(FL)+s(Chl*a*_8_) | 7 | 182.82 | 16.3 | -84.03 | 41.13 |
|  | 11 | s(FL)+s(Chl*a*_30_) | 7 | 186.1 | 18.58 | -85.67 | 36.36 |
|  | 12 | s(FL)+s(SST_3_)+S(Chl*a*_8_) | 9 | 170.76 | 4.24 | -75.76 | 43.6 |
|  | 13 | s(FL)+s(M_B50_) | 7 | 179.87 | 13.35 | -82.55 | 43.36 |
| ω**6 protists** | 1 | *Null* | 3 | 239.2 | 49.28 | -116.52 |  |
| **log(**ω**6 LC-** | 2 | s(FL) | 5 | 184.73 | 4.81 | -92.16 | 61.65 |
| **PUFA)** | 3 | s(SST_3_) | 5 | 217.40 | 27.48 | -103.50 | 55.14 |
|  | 4 | s(lat,long) | 9 | 232.06 | 42.14 | -106.40 | 61.65 |
|  | 5 | s(FL)+s(lat,long) | 11 | 205.94 | 16.02 | -91.04 | 59.3 |
|  | 6 | s(FL)+s(DOY) | 6 | 196.89 | 6.97 | -92.16 | 61.78 |
|  | 7 | s(FL)+s(lat,long)+s(DOY) | 12 | 208.29 | 18.37 | -91.04 | 59.30 |
|  | 8 | s(FL)+s(SST_3_) | 7 | 189.95 | 0.03 | -87.59 | 62.99 |
|  | 9 | s(FL)+s(SST_15_) | 7 | 192.1 | 2.18 | -88.67 | 62.97 |
|  | 10 | s(FL)+s(Chl*a*_8_) | 7 | 194.04 | 4.12 | -89.64 | 64.26 |
|  | 11 | s(FL)+s(Chl*a*_30_) | 7 | 198.91 | 8.99 | -92.07 | 62.05 |
|  | 12 | s(FL)+s(SST_3_)+S(Chl*a*_8_) | 9 | 195.51 | 5.59 | -88.13 | 73.38 |
|  | **13** | **s(FL)+s(M_B50_)** | **7** | **198.92** | **0** | **-87.58** | **72.41** |
| **Condition** | 1 | *Null* | 3 | 636.05 | 94.98 | -314.94 |  |
| **Index 1:** | 2 | s(FL) | 5 | 552.43 | 11.36 | -271.01 | 67.97 |
| ω**3/**ω**6** | 3 | s(SST_3_) | 5 | 612.04 | 70.97 | -300.82 | 62.96 |
|  | 4 | s(lat,long) | 9 | 602.89 | 61.83 | -291.82 | 58.78 |
|  | 5 | s(FL)+s(lat,long) | 11 | 552.44 | 11.38 | -264.29 | 69.27 |
|  | 6 | s(FL)+s(DOY) | 6 | 554.40 | 13.33 | -270.91 | 68.47 |
|  | 7 | s(FL)+s(lat,long)+s(DOY) | 12 | 551.29 | 10.22 | -262.52 | 69.89 |
|  | **8** | **s(FL)+s(SST_3_)** | **7** | **541.07** | **0** | **-263.49** | **70.87** |
|  | 9 | s(FL)+s(SST_15_) | 7 | 544.15 | 3.08 | -264.69 | 69.66 |
|  | 10 | s(FL)+s(Chl*a*_8_) | 7 | 553.18 | 12.12 | -269.21 | 68.83 |
|  | 11 | s(FL)+s(Chl*a*_30_) | 7 | 554.15 | 13.50 | -269.90 | 68.51 |
|  | 12 | s(FL)+s(SST_3_)+S(Chl*a*_8_) | 9 | 541.75 | 0.68 | -260.91 | 70.05 |
|  | 13 | s(FL)+s(M_B50_) | 7 | 551.89 | 10.82 | -268.56 | 70.37 |
| **Condition** | 1 | *Null* | 3 | 246.46 | 1.73 | -118.65 |  |
| **Index 2:** | **2** | **s(FL)** | **5** | **244.77** | **0** | **-119.30** | **60.26** |
| **Log(TFA %)** | 3 | s(SST_3_) | 5 | 246.72 | 1.95 | -118.16 | 58.64 |
|  | 4 | s(lat,long) | 9 | 258.33 | 15.03 | -118.54 | 57.69 |
|  | 5 | s(FL)+s(lat,long) | 11 | 259.15 | 14.39 | -117.65 | 60.44 |
|  | 6 | s(FL)+s(DOY) | 6 | 248.66 | 3.89 | -118.04 | 60.26 |
|  | 7 | s(FL)+s(lat,long)+s(DOY) | 12 | 261.51 | 16.74 | -117.65 | 60.44 |
|  | 8 | s(FL)+s(SST_3_) | 7 | 250.12 | 5.35 | -117.68 | 60.53 |
|  | 9 | s(FL)+s(SST_15_) | 7 | 249.84 | 5.08 | -117.54 | 60.96 |
|  | 10 | s(FL)+s(Chl*a*_8_) | 7 | 251.06 | 6.29 | -118.15 | 60.40 |
|  | 11 | s(FL)+s(Chl*a*_30_) | 7 | 250.76 | 5.99 | -118.00 | 60.39 |
|  | 12 | s(FL)+s(SST_3_)+S(Chl*a*_8_) | 9 | 254.46 | 9.69 | -117.60 | 60.35 |
|  | 13 | s(FL)+s(M_B50_) | 7 | 251.69 | 6.93 | -118.46 | 60.52 |
| **EPA mg/100g** | 1 | *Null* | 3 | 343.28 | 11.45 | -169.06 |  |
| **(log_10_ scaled)** | 2 | s(FL) | 5 | 335.15 | 2.33 | -162.37 | 65.07 |
|  | 3 | s(SST_3_) | 5 | 335.44 | 6.12 | -162.52 | 64.59 |
|  | 4 | s(lat,long) | 9 | 343.85 | 10.25 | -161.80 | 62.0.2 |
|  | 5 | s(FL)+s(lat,long) | 11 | 337.58 | 4.76 | -159.86 | 66.01 |
|  | 6 | s(FL)+s(DOY) | 6 | 337.32 | 4.49 | -162.37 | 65.07 |
|  | 7 | s(FL)+s(lat,long)+s(DOY) | 12 | 339.94 | 7.11 | -156.86 | 66.81 |
|  | 8 | s(FL)+s(SST_3_) | 7 | 333.10 | 0.27 | -159.16 | 65.13 |
|  | **9** | **s(FL)+s(SST_15_)** | **7** | **332.82** | **0** | **-159.03** | **66.85** |
|  | 10 | s(FL)+s(Chl*a*_8_) | 7 | 338.62 | 5.80 | -161.93 | 65.65 |
|  | 11 | s(FL)+s(Chl*a*_30_) | 7 | 336.60 | 3.78 | -160.92 | 66.21 |
|  | 12 | s(FL)+s(SST_3_)+S(Chl*a*_8_) | 9 | 336.64 | 3.82 | -158.70 | 65.56 |
|  | 13 | s(FL)+s(M_B50_) | 7 | 337.01 | 4.19 | -161.12 | 66.18 |
| **DHA mg/100g** | 1 | *Null* | 3 | 266.37 | 0.14 | -130.11 |  |
| **(log_10_ scaled)** | **2** | **s(FL)** | **5** | **266.38** | **0** | **-127.99** | **49.92** |
|  | 3 | s(SST_3_) | 5 | 269.23 | 2.85 | -129.41 | 49.47 |
|  | 4 | s(FL)+s(lat,long) | 9 | 276.16 | 10.24 | -128.46 | 50.64 |
|  | 5 | s(FL)+s(lat,long) | 11 | 281.43 | 15.06 | -128.79 | 50.85 |
|  | 6 | s(FL)+s(DOY) | 6 | 267.82 | 1.45 | -127.62 | 50.50 |
|  | 7 | s(FL)+s(lat,long)+s(DOY) | 12 | 282.13 | 15.75 | -127.96 | 51.39 |
|  | 8 | s(FL)+s(SST_3_) | 7 | 271.24 | 4.86 | -128.24 | 51.10 |
|  | 9 | s(FL)+s(SST_15_) | 7 | 271.09 | 4.72 | -128.16 | 50.88 |
|  | 10 | s(FL)+s(Chl*a*_8_) | 7 | 270.32 | 3.95 | -127.78 | 50.49 |
|  | 11 | s(FL)+s(Chl*a*_30_) | 7 | 270.05 | 3.67 | -127.64 | 20.14 |
|  | 12 | s(FL)+s(SST_3_)+S(Chl*a*_8_) | 9 | 274.56 | 8.19 | -127.66 | 50.64 |
|  | 13 | s(FL)+s(M_B50_) | 7 | 271.12 | 4.75 | -128.18 | 61.93 |

For different models types: s – smoother for parameter, FL – fork length (cm), SST_3_ and SST_15_ – sea surface temperature of a 3- or 15-day composite, Chl*a*_8_ and Chl*a*_30_ – chlorophyll a concentrations for a 8- or 30-day composite, lat – latitude, long – longitude, M_B50_ – mean phytoplankton cell size.
